# A pharmacist-led intervention for increasing the uptake of Home Medicines Review (HMR) among residents of retirement villages (PHARMER): protocol for a cluster randomised controlled trial

**DOI:** 10.1186/1472-6963-11-292

**Published:** 2011-10-31

**Authors:** Cik Yin Lee, Johnson George, Rohan A Elliott, Kay Stewart

**Affiliations:** 1 Centre for Medicine Use and Safety, Faculty of Pharmacy and Pharmaceutical Sciences, Monash University, 381 Royal Parade, Parkville, Victoria 3052, Australia; 2 Pharmacy Department, Austin Health, 145 Studley Road, P.O.Box 555, Heidelberg, Victoria 3084, Australia

## Abstract

**Background:**

The majority of retirement village residents are at risk of medication misadventure. In a recent survey of retirement village residents in Victoria, two-thirds had at least one medication-related risk factor, and hence were eligible to receive a government-subsidised Home Medicines Review (HMR). However, only 6% of eligible residents had received a HMR in the previous 12 months. Reasons for the poor uptake of HMR, and interventions for improving HMR uptake, have been identified and developed with input from stakeholders. The trial will test the effect of **P**harmacist-conducted **H**MR to **A**ddress the **R**isk of **M**edication-related **E**vents in **R**etirement Villages (PHARMER) in improving the uptake of HMRs among retirement village residents.

**Methods/Design:**

This is a multicentre prospective cluster randomised controlled trial. Ten retirement villages in Victoria, Australia will be recruited for this trial. Retirement villages will be selected in consultation with the Residents of Retirement Villages Victoria Inc. (RRVV), based on geographical locations (e.g. northeast or southwest), size and other factors. Residents from selected villages will be recruited with the help of RRVV Resident Liaison Officers using a range of strategies. Randomisation will be by geographical location to minimise contamination. Participating villages and residents will be allocated to either Pharmacist Intervention Group (PIG) or Usual Care Group (UCG). Each group will include five retirement villages and will have at least 77 residents in total. The intervention (PHARMER) comprises educating residents regarding HMR, and using a risk assessment checklist by residents to notify their General Practitioners of their medication risk. Uptake of HMR and medication adherence will be assessed in both PIG and UCG at three and six months using telephone interviews and questionnaires.

**Discussion:**

This study is the first to develop and test an intervention to improve the uptake of HMR among Australian residents in retirement villages, with a view to decreasing medication risk. A multi-faceted interventional approach will be used as suggested by stakeholders. The trial is expected to be complete by late 2011 and results will be available in 2012.

**Trial Registration:**

Australian New Zealand Clinical Trials Registry (ACTRN12611000109909)

## Background

Retirement villages offer accommodation for older people who wish to live independently or with limited assistance within a supportive community. There are approximately 1,756 retirement villages in Australia, which accommodate approximately 80,000 residents [1,2]. The number of residents in retirement villages is projected to increase to 300,000 by 2051 [2].

The majority of residents living in retirement villages are at risk of medication misadventure. In our recent survey of 2,116 retirement village residents in Australia, 47% were using multiple medications, and 65% had one or more medication-related risk factors, such as presence of three or more health conditions, use of five or more medications, use of 12 or more medication doses a day, use of medications with narrow therapeutic index or recent changes to their medication regimen [3]. On average, residents in the survey were using four medications [3]. A previous study found that 41% of retirement village residents were using multiple medications including potentially inappropriate combinations, confirming that residents in retirement villages are at risk of medication misadventure [4].

The Australian Pharmaceutical Advisory Council, the National Prescribing Service and the Pharmacy Guild of Australia currently recommend medication reviews to be conducted annually for people who are at risk of medication misadventure [5-7]. Home Medicines Review (HMR) is a government-subsidised medication management review service that aims to optimise medication use and health outcomes for individual patients living in the community [8]. There is no charge to the patient, and both general practitioners (GPs) and pharmacists are reimbursed by Commonwealth Government for providing HMR [8]. HMR is a GP-initiated service conducted by a pharmacist who is accredited to undertake medication reviews.

The 65% of residents who had at least one medication-related risk factor in our survey would meet the eligibility criteria for a HMR [3]; however, only 6% of these at-risk residents had received a pharmacist-conducted HMR in the previous 12 months, despite the fact that the majority (94%) had regular visits to their GPs [3].

The reasons for the poor uptake of HMR among retirement village residents have been investigated by our team, and interventions for increasing the uptake of HMR in this group have been developed with input from stakeholders including retirement village residents, medical, pharmacy and allied health professionals [9]. A multi-faceted interventional approach targeting residents and their health professionals, such as educating residents regarding their risk factors and benefits of HMR, and notifying GPs of residents at risk of medication misadventure using a risk assessment checklist, have been recommended by stakeholders to increase the uptake of HMR in retirement village residents [9].

The **P**harmacist-conducted **H**MR to **A**ddress the **R**isk of **M**edication-related **E**vents in **R**etirement Villages (PHARMER) trial aims to test the effectiveness of a new multi-component intervention delivered by a pharmacist to increase the uptake of HMR among retirement village residents (primary aim), and to improve residents' adherence to medications (secondary aim). The hypothesis is that residents receiving the intervention are more likely to receive a HMR compared to their counterparts receiving usual care, leading to higher HMR uptake at three and six months from baseline.

## Methods/Design

### Study design

The PHARMER trial is a multi-centre prospective cluster randomised controlled trial (RCT).

### Study setting

Ten retirement villages within Victoria, Australia will be recruited for this trial.

### Identification and selection of retirement villages

Retirement villages eligible for the study, such as those with a representative officer (also known as Resident Liaison Officer [RLO]) from the Residents of Retirement Villages Victoria Inc. (RRVV) [10], will be identified in consultation with an officer of the RRVV. Eligible retirement villages meeting the study criteria will be identified by RRVV from their member database. Using the list provided by RRVV, a convenience sample of ten retirement villages will be selected for the study based on the following factors:

• having at least 100 units;

• having at least 20 residents who are members of RRVV; and

• geographical location (northeast and southwest regions).

The two geographical locations will be used to avoid potential contamination (see cluster randomisation). The geographical locations (northeast or southwest) will be randomised to intervention group (Pharmacist Intervention Group [PIG]) or control group (Usual Care Group [UCG]). Each group will include five retirement villages and will have at least 15 km between the PIG and UCG retirement villages. Selected villages should have at least 20 RRVV members to enhance recruitment of participants.

### Recruitment of retirement villages

The RLOs of selected retirement villages will be asked to assist in recruiting their village for the study by facilitating the investigators' approach to each village manager for permission to carry out the study in their village. If a selected village's RLO is unable to assist, another retirement village will be selected and its RLO will be approached. This process will continue until 10 RLOs and their retirement villages are recruited for the trial. Written permission will be obtained from each village manager at the time of recruitment of each village.

### Cluster randomisation

Randomisation to either PIG or UCG according to geographical location (northeast or southwest) will be carried out by an independent person, using a simple randomisation technique (e.g. pick of an envelope) at the completion of retirement village recruitment. Randomisation by geographical locations of facility rather than individual residents or individual retirement villages will be used to minimise any potential contamination from sharing of information between residents belonging to different study groups, or from the same health professionals being involved in the care of residents from both groups.

### Recruitment of participants

Participants will be residents living in participating retirement villages, recruited with the help of RLOs and village managers. Residents will be recruited using a range of strategies, which include:

• distributing invitation forms to all residents in each village;

• displaying a poster on the notice board or in the common area of each village; and

• advertising the study in the villages' newsletters and/or on the villages' multi-screen televisions (where available).

Residents who are interested in the study will be asked to complete the invitation form and return it to the study investigators using a provided reply-paid envelope, or to contact the study investigators. The invitation form includes questions that ask for the resident's name and contact details, and assess their eligibility for participating in the study. Returned invitation forms will be used to identify and select residents who are eligible for the study. Eligible residents will be invited to participate in the study over telephone. Those who are interested in participating after this initial discussion will be sent a plain language statement, a consent form and a questionnaire. They will be asked to complete the consent form and questionnaire, and return them to the investigators. Once written consent is obtained, eligible residents will be enrolled in the trial and allocated to either PIG or UCG depending on the location of the retirement village they live in.

### Inclusion criteria

Residents who meet the following criteria will be eligible to participate in the study:

• living in one of the participating retirement villages;

• aged 55 years and above;

• using three or more prescribed medications;

• expecting to visit their GP in the next three months;

• expecting to be available for follow-up for at least six months from baseline; and

• available to attend an education session.

### Exclusion criteria

Residents will be ineligible to participate in the study if they have any of the following characteristics:

• received a HMR in the previous 12 months;

• planning to move from their current retirement village in the next six months;

• unable to give written informed consent; or

• unable to communicate in English.

The aim of the PHARMER trial is to target residents who have not received a HMR in the previous 12 months and improve their uptake of HMR using educational interventions. Those who have had a HMR experience in the previous 12 months are less likely to receive any additional benefits from the educational interventions because they are already aware of the benefits of HMR; and they are also less likely to receive another HMR referral from their GPs in the next 12 months, unless their condition changes (e.g. a recent hospitalisation) and requires more frequent review.

### Sample size

The sample size required for this study was calculated based on the HMR uptake in the previous 12 months (6%) reported in our recent survey [3]. From this figure, we anticipate the uptake of HMR in the UCG to be 6% after 12 months, 3% after 6 months and 1.5% after three months. To demonstrate that the intervention increases the uptake of HMRs from 3% (in the UCG) to 20% (in the PIG) at six months (considered to be a relatively conservative but clinically meaningful increase), with 80% power and a two-sided alpha value of 0.05, 55 participants will be required in each group (total of 110 participants). Allowing for 20% drop-out or loss to follow-up, and 20% for clustering effects, at least 77 participants will be recruited into each arm at baseline (total of at least 154 participants).

### Interventions in the Pharmacist Intervention Group (PIG)

At baseline, participants in the PIG will receive the intervention. The intervention to be tested includes several components:

a. HMR education session

• A pharmacist, who is accredited to undertake HMRs, will visit the PIG participants at their villages to deliver a group education session about the role of and benefits from HMR. Information leaflets about HMR will be given out to participants during these education sessions.

b. Medication risk assessment

• A medication risk assessment checklist will be adapted from a screening tool that was previously developed and published by the Pharmacy Guild of Australia (see Additional file 1: Pharmacy Guild of Australia's HMR consumer brochure). The HMR consumer brochure includes a self-administered checklist (with tick boxes) consisting of 16 items assessing a person's risk factors for medication-related problems (e.g. having recent hospitalisation, using multiple medicines, having recent changes to medication regimen, and using low therapeutic index medicines). The checklist will be completed by participants after the education session, with the pharmacist taking them through each question. The completed checklist will be used by the pharmacist to identify participants who have medication risk factors and thus could potentially benefit from a HMR.

c. New referral process for HMR

• Participants identified as having one or more medication risk factors will be referred to their GP. Referral will be made by asking the at-risk participant to give the completed medication risk checklist to their GP during their next GP visit and discuss the need for a HMR.

The group education session, including completion of the medication risk checklist, will take approximately one and a half hours.

### Routine care in the Usual Care Group (UCG)

Participants enrolled in the UCG will continue to receive routine care for six months during the study. No intervention will be offered to this group at any time during the study; however, they will not be denied a HMR if they are being offered this in the course of study as part of routine care. To ensure equity in both PIG and UCG and to avoid unfair disadvantage to any group, UCG participants will be offered a similar intervention to the PIG after the study period (e.g. at six months).

### Outcome measures and data collection

#### Outcome measures

The primary outcome measure will be participants' uptake of HMR; and secondary outcome measure will be participant self-reported medication adherence. HMR uptake will be assessed at three and six months from baseline, and medication adherence at six months.

#### Baseline data collection

The baseline characteristics of participants will be collected using a self-completed questionnaire. At baseline, the questionnaire will be sent to participants with the plain language statement and consent form. Information collected by the baseline questionnaire includes:

• demographics;

• medication use data, including medication management and problems experienced with medications;

• medication adherence [11];

• health-related quality of life [12]; and

• medication-related risk factors and eligibility for HMR (see Additional file 1: Pharmacy Guild of Australia's HMR consumer brochure).

Participants' medication adherence will be assessed using the self-reported Morisky scale [11]. The Morisky scale comprises four validated items, with responses to each item scored 0 for 'yes' and 1 for 'no' (except for the item 'Are you always careful in taking your medicines?' where the score is reversed). The total score on the Morisky scale ranges between 0 and 4; a score of less than 4 represents nonadherence.

Participants' health-related quality of life will be assessed using the self-reported Short Form (SF)-12^® ^Health Survey [12]. The SF-12^® ^Health Survey consists of 12 validated items relating to physical and mental health function. Responses to the items will be scored and computed using an online scoring program [13]. The scoring method will give two sets of results - Physical Component Summary (PCS) and Mental Component Summary (MCS) scores, with the scores ranging from 0 to 100, zero representing the lowest level of health and 100 representing the highest level of health [14].

Participants in both groups will be assessed for their medication-related risk factors and eligibility for receiving a HMR using the criteria or items in the Pharmacy Guild of Australia's HMR consumer brochure (see Additional file 1). Participants who fulfil one or more of these criteria will be considered eligible for a HMR.

Participants will be contacted by telephone to obtain incomplete or missing responses.

### Three-month follow up

At three months from baseline, participants in both the PIG and the UCG will be interviewed by telephone to assess their HMR uptake. In addition, participants in the PIG will also be assessed for their experience of the pharmacist-education session about the HMR service. Participants' uptake of HMR will be measured as the proportion of participants who have had at least one HMR since baseline.

### Six-month follow up

At six months from baseline, measures of uptake of HMR will be repeated in participants who have not received a HMR at three months. To ensure reliability, participant self-reported uptake of HMR will be verified with their GP, community pharmacist and/or the pharmacist who undertook the HMR, where possible. The HMR uptake at six months from baseline will be compared between the two study groups.

Participants in both groups will also be asked to complete a questionnaire. Information collected at the six-month follow up will include:

• any changes to medication regimens, including problems experienced with use of medications since baseline;

• medication adherence [11]; and

• health-related quality of life [12].

Data at three and six months will be collected by an independent person who will be blinded to the group allocation.

### Data analysis

All data will be analysed according to intention-to-treat principles. The baseline characteristics of participants in the PIG and UCG will be compared using Chi-square, Mann-Whitney U or Student t-test. Uptake of HMR in the two study groups at three and six months will be compared using Chi-square test. Paired samples t-test or Wilcoxon Signed-Rank test will be used to compare changes in Morisky scores and SF-12^® ^Health Survey scores between the two groups at six months.

### Ethics

This study has been approved by the Monash University Human Research Ethics Committee. Written informed consent will be obtained from all RLOs and participants at the time of enrolment.

### Study timeline

Ethics approval: December 2010

Trial commencement: January 2011 (recruitment of RLOs and retirement villages)

Recruitment of participants and baseline data collection: February - March 2011

Implementation of interventions: April - June 2011

Three-month follow up: September 2011

Six-month follow up: December 2011

Data analysis: January - February 2012

## Discussion

To our knowledge this will be the first study to develop and test an intervention to improve the uptake of medication review services by retirement village residents in Australia, with a view to decreasing medication risk. Most previous research on medication review uptake has focused on the general populations living in the community or war veterans [15-17]. We will use a multi-faceted interventional approach targeting residents and their health professionals for increasing the uptake of HMR, as suggested by stakeholders. The effectiveness of such interventions (patient education and GP feedback) in increasing the uptake of HMRs has been well-established [16,18]. Our study is endorsed and supported by RRVV and the Council on the Ageing Victoria (COTA). Such collaborations with RRVV and COTA, including the input from RLOs, will facilitate the recruitment and conduct of this study.

## List of abbreviations

HMR(s): Home Medicines Review(s); RLOs: Resident Liaison Officers; RRVV: Residents of Retirement Villages Victoria Inc.; COTA: Council on the Ageing Victoria; GP(s): general practitioner(s); RCT: Randomised Controlled Trial; PIG: Pharmacist Intervention Group; UCG: Usual Care Group; PCS: Physical Component Summary; MCS: Mental Component Summary; SF−12^® ^Health survey: Short Form−12^® ^Health Survey.

## Competing interests

The authors declare that they have no competing interests.

## Authors' contributions

CYL (PhD candidate) participated in the design of the trial, recruitment and manuscript preparation, and has an ongoing role in carrying out the trial. JG, RAE and KS participated in the design of the trial and study methodology, and review of the manuscript. All authors read and approved the final manuscript.

## Pre-publication history

The pre-publication history for this paper can be accessed here:

http://www.biomedcentral.com/1472-6963/11/292/prepub

## Supplementary Material

Additional file 1 **Pharmacy Guild of Australia's HMR consumer brochure (PDF)**. Click here for file
