# Supplementary material for: A pharmacist-led intervention for increasing the uptake of Home Medicines Review (HMR) among residents of retirement villages (PHARMER): protocol for a cluster randomised controlled trial
Source: BMC Health Serv Res. 2011 Oct 31;11:292. doi: 10.1186/1472-6963-11-292 (PMC3215972; doi:10.1186/1472-6963-11-292)
Supplement: Additional file 1 — Pharmacy Guild of Australia's HMR consumer brochure (PDF). [file 1472-6963-11-292-S1.PDF]

HMR

Get the best out of your medicines.

Ask your doctor or pharmacist about a Home Medicines Review.

For further information visit: www.guild.org.au

Australian Government
Department of Health and Ageing

The Pharmacy Guild of Australia

This document was published by the Pharmacy Guild of Australia. The Home Medicines Review (HMR) Program is funded by the Australian Government Department of Health and Ageing as part of the Community Pharmacy Agreement.

00079

HMR

Home Medicines Review

Do your medicines need a check up?

Get the best out of your medicines.

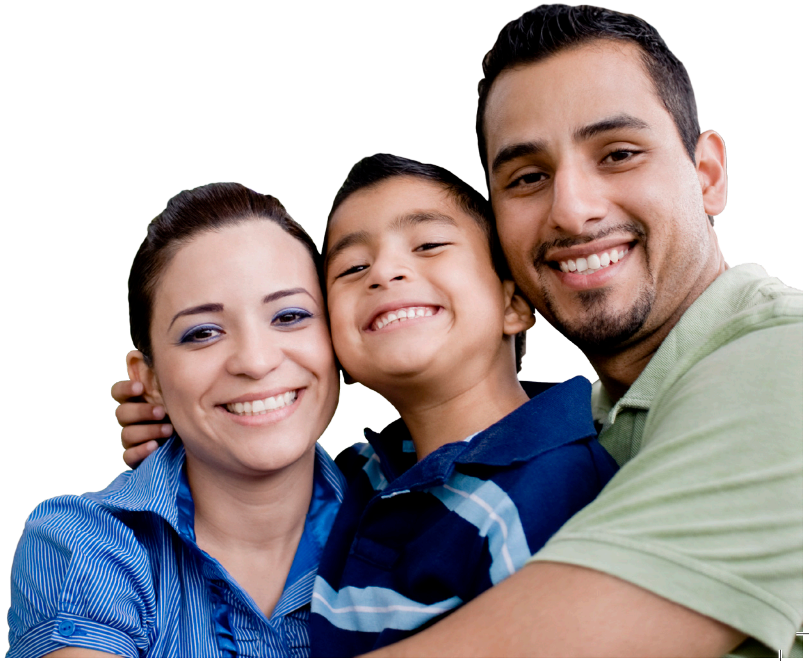

HMR

Would you or someone you care for benefit from a Home Medicines Review? ... Take the test ✓

Are your medicines right for you?

Every year, up to 210,000 Australians are admitted to hospital due to medication problems. At least 50% of these admissions could be avoided by better medicine management.

A Home Medicines Review (HMR) helps you understand your medicines and stay out of hospital.

What is a Home Medicines Review?

A Home Medicines Review is a free health service funded by the Commonwealth Government.

A Home Medicines Review involves a pharmacist visiting you at home and reviewing all your medicines. The pharmacist then works together with your doctor to develop a plan to manage and record the medicines you take.

By working together with you, your doctor and pharmacist can identify any medication problems and make sure your medicines are:

✓ The best treatment option for you;

✓ Suitable and safe to take together; and

✓ Taken correctly.

Why have a Home Medicines Review?

A Home Medicines Review will help you better manage your medicines. It will help:

✓ Increase your knowledge about your medicines;

✓ Increase your confidence in using your medicines; and

✓ Reduce your risk of an avoidable trip to hospital.

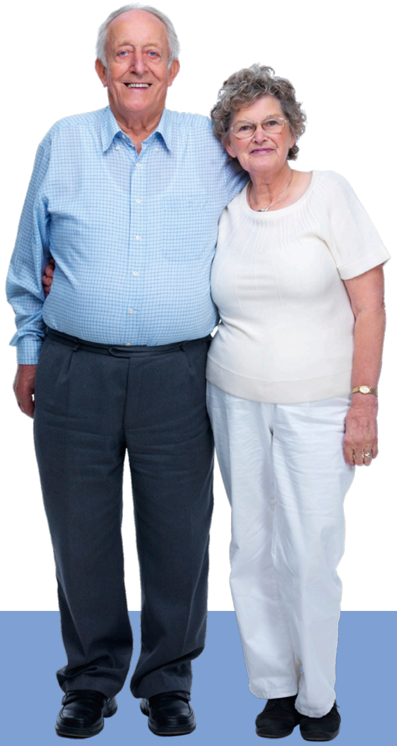

Ask your doctor or pharmacist about a Home Medicines Review.

Tick the boxes that apply:

- ☐ Have you recently been discharged from hospital?
- ☐ Are you taking several medicines? (including supermarket or herbal medicines)
- ☐ Have you had recent changes to your medicines?
- ☐ Do your medicines need monitoring? (e.g. blood thinning medicines)
- ☐ Do any of your medicines make you feel unwell?
- ☐ Do you use devices to assist with medication management such as monitoring blood glucose or a nebuliser?
- ☐ Do you attend more than one doctor including general practitioners and specialists?
- ☐ Are you sometimes unsure about which medicines you should be taking?
- ☐ Would you like to be more confident about understanding your medicines?

Many different people are helped by a Home Medicines Review.

- ☐ Are you a child or adolescent with an ongoing health condition? (e.g. asthma)
- ☐ Are you from a non-English speaking background?
- ☐ Are you receiving palliative care?
- ☐ Do you have a heart condition?
- ☐ Do you have asthma or emphysema?
- ☐ Do you have a mental health condition?
- ☐ Do you have diabetes?

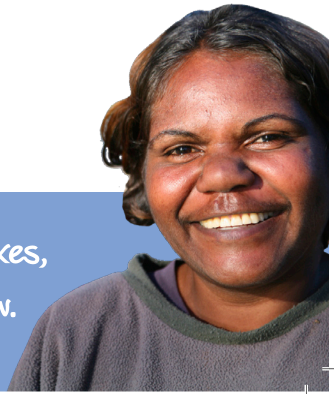

If you have ticked one or more of the above boxes, you may benefit from a Home Medicines Review.
